# The glutamate transport inhibitor DL-Threo-β-Benzyloxyaspartic acid (DL-TBOA) differentially affects SN38- and oxaliplatin-induced death of drug-resistant colorectal cancer cells

**DOI:** 10.1186/s12885-015-1405-8

**Published:** 2015-05-16

**Authors:** Elena Pedraz-Cuesta, Sandra Christensen, Anders A. Jensen, Niels Frank Jensen, Lennart Bunch, Maria Unni Romer, Nils Brünner, Jan Stenvang, Stine Falsig Pedersen

**Affiliations:** 1 Department of Biology, Faculty of Science, University of Copenhagen, 13, Universitetsparken, DK-2100 Copenhagen, Denmark; 2 Department of Drug Design and Pharmacology, Faculty of Health and Medical Sciences, 13, Universitetsparken, DK-2100 Copenhagen, Denmark; 3 Faculty of Health and Medical Sciences, Institute of Veterinary Disease Biology, University of Copenhagen, Copenhagen, Denmark; 4 Department of Clinical Physiology, Nuclear Medicine and PET, Rigshospitalet, University of Copenhagen, Copenhagen, Denmark

**Keywords:** SLC1A1, EAAT3, SLC1A3, EAAT1, GSH, Glutathione, LoVo, HCT116, Irinotecan

## Abstract

**Background:**

Colorectal cancer (CRC) is a leading cause of cancer death globally and new biomarkers and treatments are severely needed.

**Methods:**

Here, we employed HCT116 and LoVo human CRC cells made resistant to either SN38 or oxaliplatin, to investigate whether altered expression of the high affinity glutamate transporters Solute Carrier (SLC)-1A1 and -1A3 (EAAT3, EAAT1) is associated with the resistant phenotypes. Analyses included real-time quantitative PCR, immunoblotting and immunofluorescence analyses, radioactive tracer flux measurements, and biochemical analyses of cell viability and glutathione content. Results were evaluated using one- and two-way ANOVA and Students two-tailed *t-*test, as relevant.

**Results:**

In SN38-resistant HCT116 and LoVo cells, SLC1A1 expression was down-regulated ~60 % and up-regulated ~4-fold, respectively, at both mRNA and protein level, whereas SLC1A3 protein was undetectable. The changes in SLC1A1 expression were accompanied by parallel changes in DL-Threo-β-Benzyloxyaspartic acid (TBOA)-sensitive, UCPH101-insensitive [^3^H]-D-Aspartate uptake, consistent with increased activity of SLC1A1 (or other family members), yet not of SLC1A3. DL-TBOA co-treatment concentration-dependently augmented loss of cell viability induced by SN38, while strongly counteracting that induced by oxaliplatin, in both HCT116 and LoVo cells. This reflected neither altered expression of the oxaliplatin transporter Cu^2+^-transporter-1 (CTR1), nor changes in cellular reduced glutathione (GSH), although HCT116 cell resistance per se correlated with increased cellular GSH. DL-TBOA did not significantly alter cellular levels of p21, cleaved PARP-1, or phospho-Retinoblastoma protein, yet altered SLC1A1 subcellular localization, and reduced chemotherapy-induced p53 induction.

**Conclusions:**

SLC1A1 expression and glutamate transporter activity are altered in SN38-resistant CRC cells. Importantly, the non-selective glutamate transporter inhibitor DL-TBOA reduces chemotherapy-induced p53 induction and augments CRC cell death induced by SN38, while attenuating that induced by oxaliplatin. These findings may point to novel treatment options in treatment-resistant CRC.

**Electronic supplementary material:**

The online version of this article (doi:10.1186/s12885-015-1405-8) contains supplementary material, which is available to authorized users.

## Background

Colorectal cancer (CRC) is the fourth most common cause of cancer death worldwide [1, 2]. Currently, treatment of CRC is based on combination of 5-fluorouracil (5-FU) and leucovorin [3–5] with other chemotherapeutic drugs. In addition, despite frequent resistance development, targeted treatment with the epidermal growth factor receptor (EGFR) inhibitor cetuximab or the angiogenesis-inhibitory antibody bevacizumab is successful in some patients [4]. The combination treatments FOLFOX (5-FU + leucovorin + oxaliplatin) [6] and FOLFIRI (5-FU + leucovorin + irinotecan) [7] significantly prolong progression-free survival in advanced CRC, the choice between irinotecan and oxaliplatin being largely dictated by toxicity issues [8]. Oxaliplatin is a diaminocyclohexane platinum derivative which induces formation of DNA adducts, and irinotecan is the precursor of the topoisomerase-I inhibitor 7-ethyl-10-hydroxycamptothecin (SN38). Both compounds induce DNA damage, upregulation of p53 and p21^WAF1/Cip1^, cell cycle arrest, and cell death [9–11]. The majority of patients with metastatic CRC, whether on FOLFOX or FOLFIRI, will experience treatment resistance and disease progression upon treatment, leaving only limited additional treatment options. Possible remedies to this include the development of drugs that do not exhibit cross-resistance with those currently used, and of predictive biomarkers ensuring that patients receive the treatment with the highest likelihood of effect [5]. Although progress has been made in recent years, strong biomarkers predicting response to oxaliplatin or irinotecan are lacking and urgently needed [3, 4, 12].

To gain insight into the molecular mechanisms underlying chemotherapy resistance, we developed drug-resistant human CRC cell lines based on the well-characterized HCT116 and LoVo cell lines. Sublines resistant to SN38 and oxaliplatin, respectively, were established by long-term exposure to increasing doses of these drugs. The cell lines developed exhibit little cross-resistance between SN38 and oxaliplatin [13]. Microarray analyses demonstrated marked changes in mRNA profiles of resistant cells compared to their parental counterparts. Among these, we noted major changes in mRNA levels of the high affinity excitatory amino acid transporters (or glutamate transporters) Solute Carrier (SLC) 1A1 and -1A3 (EAAT3 and EAAT1, respectively), in the resistant cell lines [13]. Studies of plasma membrane transport proteins in chemotherapy-resistant tumor cells have generally focused on ABC-transporters [14, 15]. However, a number of properties make the SLC1A family (SLC1A1-A7) very interesting in this context. Although some isoforms, including SLC1A1 and SLC1A3, are also found in peripheral tissues, the SLC1A family is by far most widely expressed in the brain [16–18]. SLC1A family transporters mediate cellular uptake of glutamate, driven by 3Na^+^,1H^+^ cotransport, 1 K^+^ counter-transport. In addition, SLC1A1 has high capacity for transporting l-cysteine, a precursor in glutathione synthesis [16]. *Slc1a1* and *Slc1a3* knockout mice show retinal ganglion cell degeneration, altered brain glutamate homeostasis, and increased oxidative stress sensitivity [19], and *Slc1a1* knockout mice exhibit brain atrophy and reduced neuronal levels of the antioxidant tripeptide (glutamate, cysteine, glycine) glutathione [20], consistent with a role for these transporters in glutathione synthesis. A few studies reported altered expression and localization of glutamate transporters in CNS [21] and non-CNS [18] cancers. Gliomas down-regulate SLC1A family transporters and switch from net uptake to net efflux of glutamate. This stimulates their growth and motility in an autocrine fashion, while exerting toxic effects on surrounding neurons [21–23]. Furthermore, increased levels of reduced glutathione (GSH) have been associated with chemotherapy resistance in several cancer types [24]. However, the possible role of glutamate transporters in CRC chemotherapy resistance has, to our knowledge, never been addressed.

The aim of this study was to investigate the regulation and possible roles of glutamate transporters SLC1A1 and SLC1A3 in SN38- and oxaliplatin-resistance in CRC. We show that SLC1A1 expression and glutamate transporter activity are altered in a parallel manner in SN38-resistant CRC cells. The glutamate transporter inhibitor DL-TBOA reduces chemotherapy-induced p53 induction and augments CRC cell death induced by SN38, while strongly attenuating that induced by oxaliplatin. Collectively, our findings indicate that changes in glutamate transporter expression and activity may be relevant to the prediction and treatment of CRC chemotherapy resistance, and that cotreatment with DL-TBOA may be beneficial in combination with irinotecan, but detrimental in combination with oxaliplatin treatment.

Part of this work has previously been reported in abstract form [25].

## Results

### Expression and activity of glutamate transporters are altered in resistant CRC cells

Our recent microarray analysis pointed to robust changes in the expression of glutamate transporters SLC1A1 and SLC1A3 upon resistance development in both HCT116 cells and LoVo cells (Additional file 1: Figure S1A) [13]. Strikingly, analysis of publically available CRC patient tissue data (www.oncomine.org; [26]) showed a significant down-regulation of SLC1A1 mRNA levels in CRC compared to normal tissue in 11 out of 15 datasets, while SLC1A3 expression was generally unaltered (Additional file 1: Figure S1B).

**Fig. 1:**
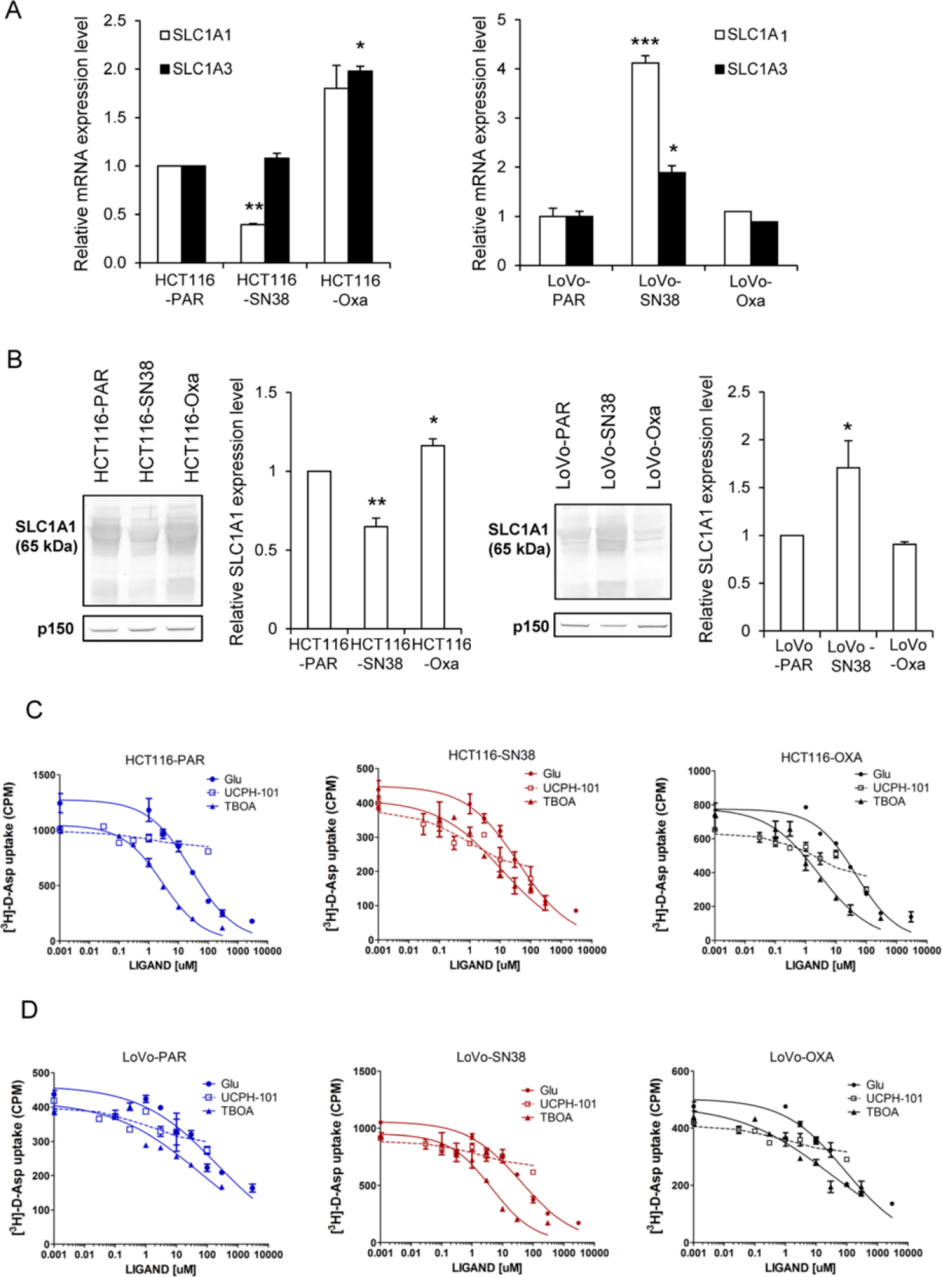
Expression and activity of SLC1A1 and SLC1A3 is altered in SN38- and oxaliplatin-resistant CRC lines. **a** Relative mRNA levels of SLC1A1 and SLC1A3 in parental (PAR), SN38- and oxaliplatin-resistant HCT116 and LoVo cells, determined by qPCR analysis. **b** Protein levels of SLC1A1 in parental, SN38- and oxaliplatin-resistant HCT116 and LoVo cells relative to that in their parental counterparts. Representative Western blots (p150 serves as a loading control) and densitometric quantification of the Western blot data are shown. The qPCR and Western blot data represent 3 independent experiments per condition. *) *p* < 0.05, **) *p* < 0.01, and ***) *p* < 0.001, compared to parental cells by one-way ANOVA and Dunnett post-test. **c-d** [^3^H]-D-Asp uptake level in parental (PAR), SN38- and oxaliplatin-resistant HCT116 and LoVo cells in the [^3^H]-D-Asp uptake assay. Concentration-inhibition curves for L-Glutamate (L-Glu), DL-TBOA (TBOA) and UCPH-101 in parental, SN38- and oxaliplatin-resistant HCT116 and LoVo cells, respectively. Values are based on four experiments each performed in duplicate

We therefore asked whether changes in SLC1A1 and SLC1A3 expression were involved in resistance development in HCT116 and LoVo cells. Consistent with the microarray data, qPCR analysis showed that the SLC1A1 mRNA level was down-regulated in HCT116-SN38 cells compared to that in parental cells (Fig. 1a). The SLC1A3 mRNA level was increased in oxaliplatin-resistant HCT116 cells and unaffected in SN38-resistant HCT116 cells. In LoVo cells, both SLC1A1 and SLC1A3 mRNA levels were increased in SN38-resistant cells and unaffected in oxaliplatin-resistant cells, compared to the levels in parental cells (Fig. 1a).

Protein levels of SLC1A1 followed the same pattern as the mRNA levels, i.e. SLC1A1 protein expression was down-regulated in SN38-resistant HCT116 cells, and increased in oxaliplatin-resistant HCT116 cells and SN38-resistant LoVo cells, compared to parental levels (Fig. 1b). For SLC1A3, no protein band of the expected size was detectable for either of the reported splice variants (~60 and ~55 kDa) [27], using 3 different antibodies which all gave clear bands of correct size in positive control mouse brain tissue (not shown). Although other scenarios are possible, this suggests that the SLC1A3 protein level is very low in CRC cells.

As glutamate transporter activity and membrane localization are heavily posttranslationally regulated [28], expression levels alone do not reveal whether transport activity is altered. We therefore next determined glutamate transporter activity (as uptake of the substrate [^3^H]-D-Asp following a 6-min incubation in buffer supplemented with a tracer concentration of 100 nM [^3^H]-D-Asp). Data are shown in Fig. 1c, d and Table 1. In parental HCT116 and LoVo cells, [^3^H]-D-Asp uptake was competitively inhibited by the substrate L-glutamate, with IC_50_ values of 20–30 μM. To determine which transporter(s) was responsible for the [^3^H]-D-Asp uptake, we assessed the effect of DL-TBOA, a nonselective inhibitor of EAATs, and UCPH-101, a specific SLC1A3 inhibitor [16, 28, 29]. IC_50_ values of DL-TBOA for SLC1A1 and SLC1A3 in uptake assays are in the low micromolar range, depending on the system and experimental setup [30, 31], whereas UCPH-101 exhibits high-nanomolar IC_50_ values for SLC1A3 and is inactive at SLC1A1 at concentration up to > 400 fold higher [29]. In all cell lines, basal [^3^H]-D-Asp uptake was inhibited by DL-TBOA with IC_50_ values around 2 μM, whereas it was essentially unaffected by UCPH-101 at concentrations up to 100 μM. Basal [^3^H]-D-Asp uptake was decreased by about 60 % in SN38-resistant compared to parental HCT116 cells, whereas that in SN38-resistant LoVo cells was nearly tripled compared to parental LoVo cells. In the oxaliplatin-resistant cell lines, [^3^H]-D-Asp uptake was slightly decreased in the HCT116 model, and unaltered in the LoVo model.

**Table 1:** Summary of pharmacological properties and basal level [^3^H]-D-Asp uptake

	Substrate/Inhibitor			
cell line	L-Glu (μM) IC_50_[pIC_50_ ± S.E.M.]	UCPH (μM) IC_50_[pIC_50_ ± S.E.M.]	TBOA (μM) IC_50_[pIC_50_ ± S.E.M.]	Basal Uptake [% of parental]
HCT116-PAR	21 [4.67 ± 0.04]	>100 [<4.0]	1.8 [5.75 ± 0.03]	100
HCT116-SN38	21 [4.67 ± 0.01]	>100 [<4.0]	1.5 [5.83 ± 0.08]	40 ± 1.8***
HCT116-Oxa	21 [4.67 ± 0.01]	>100 [<4.0]	1.5 [5.75 ± 0.08]	73 ± 6.9*
LoVo-PAR	26 [4.59 ± 0.04]	>100 [<4.0]	1.4 [5.84 ± 0.03]	100
LoVo-SN38	28 [4.54 ± 0.10]	>100 [<4.0]	1.9 [5.83 ± 0.11]	275 ± 35**
LoVo-Oxa	23 [4.64 ± 0.06]	>100 [<4.0]	2.7 [5.75 ± 0.03]	97 ± 8.0 NS

IC_50_ values for the three compounds are in μM, with pIC_50_ values in brakets. The basal [^3^H]-D-Asp uptake data are based on the measured uptake in the chemotherapeutic-cells normalized to that in the relevant parental cell line on the experiment performed in duplicate. *) *p* <0.05,**) *p* <0.01,and ***) *p* <0.001, Compared to parental cell by two-tailed Student’s *t-*test

Collectively, these data show that SLC1A1 mRNA and protein expression and DL-TBOA-sensitive, UCPH-101-insensitive [^3^H]-D-Asp uptake are decreased in SN38-resistant HCT116 cells and increased in SN38-resistant LoVo cells, compared to their parental controls, while neither SLC1A3 protein or activity could be detected in any of the cell lines.

### Viability of SN38- and oxaliplatin-resistant CRC cells is differentially affected by DL-TBOA

**Fig. 2:**
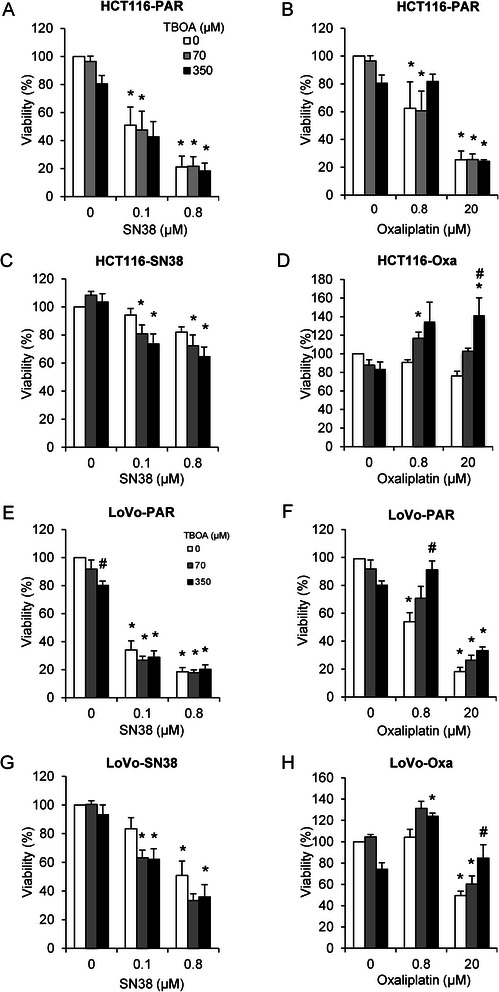
DL-TBOA augments SN38-induced death in SN38-resistant cells, but protects oxaliplatin-resistant cells from oxaliplatin-induced death. Parental and drug-resistant HCT116 and LoVo cell lines seeded in 96-well dishes were exposed to SN38 (0.1 or 0.8 μM) or oxaliplatin (0.8 or 20 μM), alone or in combination with 70 or 350 μM DL-TBOA as indicated, for 48 h. Viability was assessed by MTT assay. **a-b** Parental HCT116 cells, (**c**) SN38 resistant HCT116 cells, (**d**) Oxaliplatin-resistant HCT116 cells, (**e-f**) Parental LoVo cells, (**g**) SN38 resistant LoVo cells, (**h**) Oxaliplatin-resistant LoVo cells. Data are means with S.E.M. error bars of 3 independent experiments. *) *p* < 0.05, **) *p* < 0.01, ***) *p* < 0.001 compared to the control group without drug or TBOA treatment; #) *p* < 0.05 compared to controls without TBOA treatment. One-way ANOVA followed by Dunnett post-test

To determine whether glutamate transporter activity contributed to the SN38- and oxaliplatin-resistant phenotypes, we next assessed viability, first by MTT assay (Fig. 2). Viability of parental HCT116 (Fig. 2a, b) and LoVo (Fig. 2e, f) cell lines was reduced after 48 h exposure to SN38 or oxaliplatin, with about 20 % viable cells remaining after 48 h at the highest dose tested (0.8 μM SN38 or 20 μM oxaliplatin, respectively). Addition of DL-TBOA (70 or 350 μM) concomitantly with the chemotherapeutic drugs if anything slightly exacerbated the SN38-induced loss of viability in parental cell lines (Fig. 2a, e). In contrast, DL-TBOA counteracted the effect of oxaliplatin on viability in both parental cell lines (Fig. 2b, f). This was particularly evident in LoVo cells, in which 350 μM DL-TBOA essentially abolished the loss of viability induced by 0.8 μM oxaliplatin (Fig. 2f). Notably, the DL-TBOA-induced increase in viability was specific to oxaliplatin-treated cells, as untreated cells consistently showed a small decrease in viability upon DL-TBOA treatment (Fig. 2a-h).

We next determined whether SN38- and oxaliplatin-resistance was associated with changes in the impact of DL-TBOA on viability. Indeed, in SN38-resistant HCT116 (Fig. 2c) and LoVo (Fig. 2g) cells, concomitant DL-TBOA treatment concentration-dependently enhanced SN38-induced loss of viability. Conversely, in oxaliplatin-resistant HCT116 (Fig. 2d) and LoVo (Fig. 2h) cells, DL-TBOA reversed oxaliplatin-induced loss of viability. The MTT assay measures mitochondrial conversion of tetrazolium salt to formazan [32]. Although this is generally a good measure of cell viability, artifacts can arise if mitochondrial activity changes without parallel changes in viability. To determine viability by an independent method we therefore DAPI-labeled nuclei and quantified the surviving, still adherent cells by high-throughput confocal microscopy. The opposite effects of DL-TBOA on SN38- and oxaliplatin-induced loss of viability are also evident in this assay, strongly indicating that the effects of DL-TBOA primarily reflect changes in cell viability (Additional file 2: Figure S2).

Taken together, this data shows that DL-TBOA enhances SN38-induced, and counteracts oxaliplatin-induced, cell death.

### Expression of the Cu^2+^ transporter CTR1 is unaffected by DL-TBOA

**Fig. 3:**
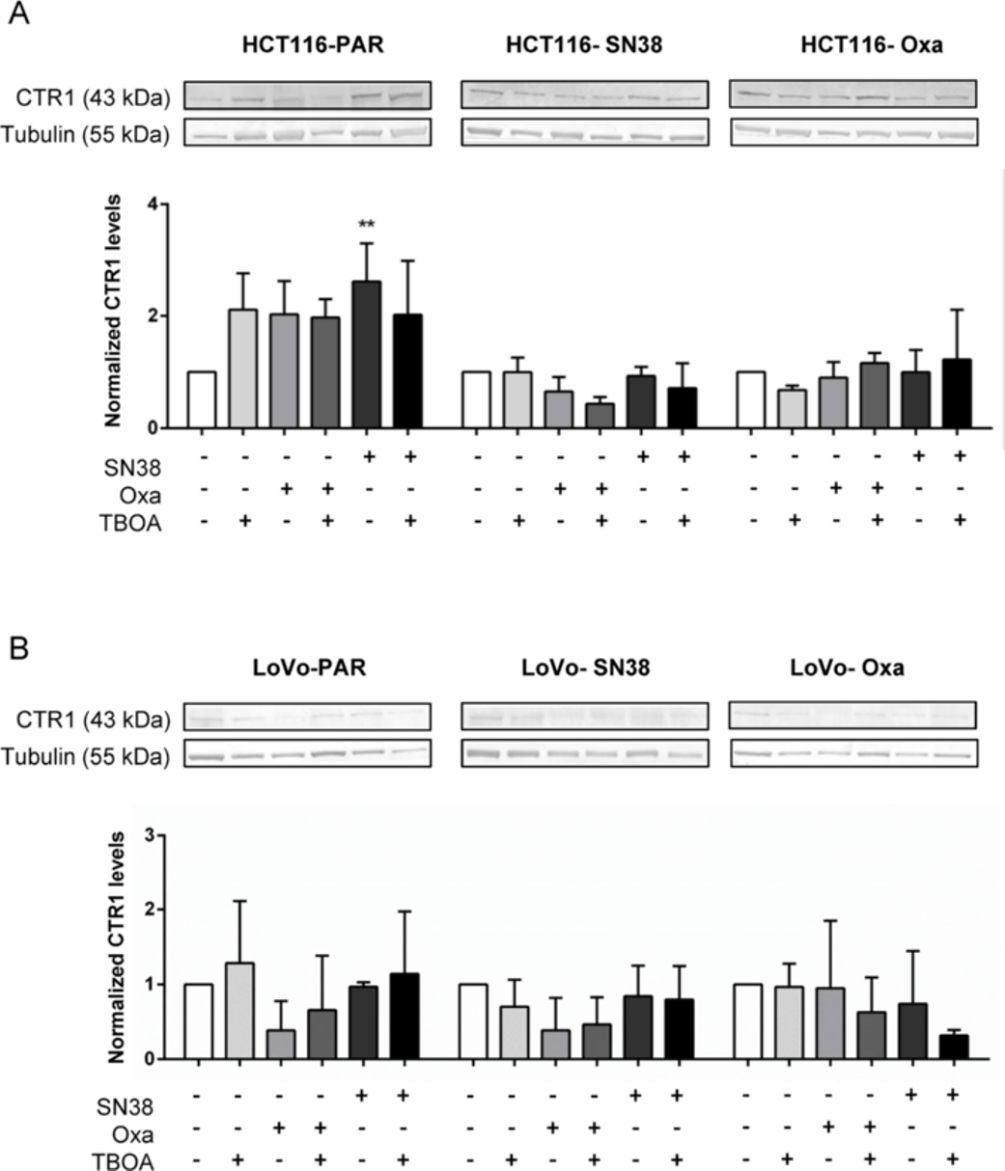
Effect of acute DL-TBOA and chemotherapy treatment on CTR1 protein level. Parental and drug-resistant HCT116 (**a**) and LoVo (**b**) cell lines were exposed to SN38 (0.8 μM) or oxaliplatin (20 μM), alone or in combination with 350 μM DL-TBOA as indicated for 24 h. Equal amounts of protein per lane were separated by SDS-PAGE and the protein levels of CTR1 were determined by Western blotting. Top: Representative Western blots (tubulin serves as a loading control), bottom: Densitometric quantification data summarized from 3 independent experiments per condition. Quantitative data are means with S.E.M. error bars of 3 independent experiments. *) *p* < 0.05, **) *p* < 0.01, ***) *p* < 0.001 compared to the control group without drug or TBOA treatment. Two-way ANOVA with Tukey post-test

The marked and specific reversal of oxaliplatin-induced cell death by DL-TBOA suggested that an oxaliplatin import mechanism might be inhibited by DL-TBOA. The high-affinity Cu^2+^ transporter CTR1 is a major such pathway [33]. We therefore hypothesized that DL-TBOA-induced rescue of CRC cells from oxaliplatin-induced death might reflect CTR1 down-regulation. To avoid confounding effects of the substantial death induction seen at 48 h, CTR1 levels were assessed after 24 h of chemotherapy +/− DL-TBOA. Oxaliplatin treatment tended to reduce CTR1 protein expression in all cell lines except parental HCT116, HCT116-Oxa, and LoVo-Oxa cells, yet without detectable effects of DL-TBOA on the CTR1 protein level (Fig. 3).

### Cellular GSH is increased in resistant HCT116 cells, but only marginally affected by DL-TBOA

**Fig. 4:**
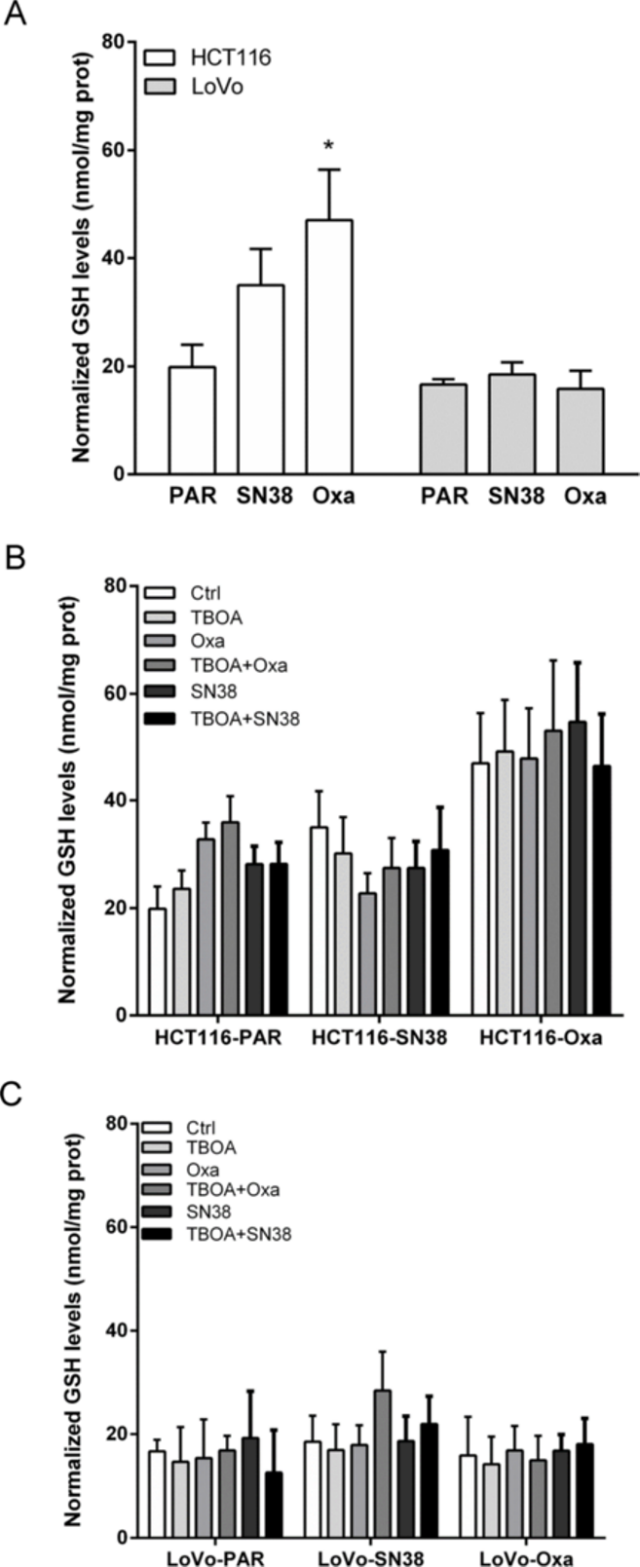
Effect of glutamate transporter inhibition on cellular GSH levels. **a** Basal intracellular GSH levels were measured as described in Materials and Methods, and normalized to total protein in the samples. **b-c** Normalized basal GSH levels under control conditions, in HCT116 and LoVo parental and resistant cells after 24 h of treatment with 0.8 μM SN38 or 20 μM oxaliplatin, in absence or presence of DL-TBOA (350 μM) as shown. * *p* < 0.05 vs untreated control group (n = 5). One-way ANOVA followed by Dunnett post-test

In light of the importance of SLC1A1 in regulation of L-cysteine transport and cellular GSH homeostasis [16, 19, 20] and the role of increased GSH levels in chemotherapy resistance in several cancer types [24], we next asked whether resistance development and DL-TBOA treatment were associated with changes in cellular GSH level. Notably, the steady state intracellular GSH level was increased in both SN38- and oxaliplatin-resistant HCT116 cells, yet unaltered in the resistant LoVo strains (Fig. 4a). After a 24 h treatment with SN38 or oxaliplatin, parental HCT116 cells showed slightly increased GSH levels, and a trend towards decreased GSH levels was seen in SN38 resistant cells (Fig. 4b). In contrast, oxaliplatin-resistant HCT116 cells (Fig. 4b) and all LoVo cell lines (Fig. 4c) showed no detectable changes in cellular GSH levels upon treatment. There was no detectable effect of DL-TBOA on GSH levels.

### p53 induction by SN38 and oxaliplatin is decreased by DL-TBOA

**Fig. 5:**
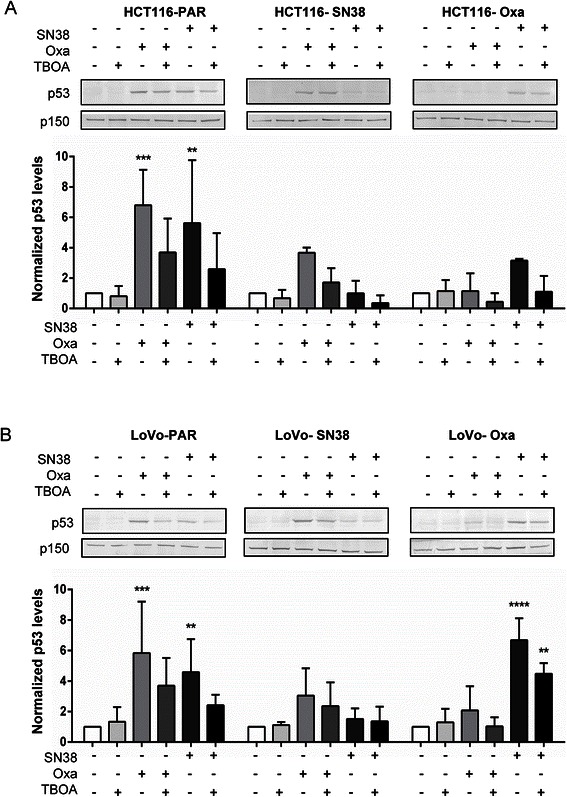
Effects of DL-TBOA on cell death and survival parameters after chemotherapy treatment. Parental and drug-resistant HCT116 (**a**) and LoVo (**b**) cell lines were exposed to SN38 (0.8 μM) or oxaliplatin (20 μM), alone or in combination with 350 μM DL-TBOA as indicated, for 24 h. Equal amounts of protein per lane were separated by SDS-PAGE and the protein level of p53 was determined by Western blotting. Top: Representative Western blots, with p150 as loading control. Bottom: Densitometric quantifications based on 3 independent experiments per condition. Data are means with S.E.M. error bars of 3 independent experiments. *) *p* < 0.05, **) *p* < 0.01, ***) *p* < 0.001,****) *p* < 0.0001 compared to the control group without drug or TBOA treatment; Two-way ANOVA with Tukey post-test

We next explored the impact of SN38, oxaliplatin and DL-TBOA on protein levels of p53 and p21^WAF1/Cip^ (p21), major cell survival- and proliferation regulators induced by DNA damage after SN38 and oxaliplatin treatment [9–11], and on PARP-1 cleavage, a well-characterized indicator of apoptosis induction. In parental HCT116 cells, p53 and p21 were markedly induced by 24 h treatment with SN38 or oxaliplatin (Fig. 5a and Additional file 3: Figure S3), consistent with the known DNA damage induction by both drugs [9–11]. In SN38-resistant HCT116 cells, this response to oxaliplatin was retained, while, as expected, SN38 had essentially no effect on p53 expression, yet modestly increased p21 expression. Conversely, in oxaliplatin-resistant cells, only SN38 induced p53 and p21 expression (Fig. 5a and Additional file 3: Figure S3). PARP-1 cleavage was induced by SN38 in parental and oxaliplatin-resistant, yet not in SN38-resistant, cells (Additional file 3: Figure S3). A comparable pattern was seen for the LoVo cell lines (Fig. 5b and Additional file 4: Figure S4). Notably, treatment with DL-TBOA concomitant to the chemotherapeutic compounds induced an apparent decrease in p53 induction compared to chemotherapy alone, in both parental and drug-resistant cell lines (Fig. 5a, b). As p53 affects both proliferation and death pathways, we next asked whether DL-TBOA affected proliferation, using retinoblastoma protein phosphorylation on Ser 807/811 (pRb) as a well-established marker of active cell cycling (Additional file 5: Figure S5). In the resistant (but, unexpectedly not in the parental), cell lines, the pRb level was decreased by the chemotherapy treatment to which the cell lines were sensitive, confirming that the treatments impact on proliferation. While these effects did not reach statistical significance, DL-TBOA also tended to increase pRb levels under control conditions in both parental cell lines, and slightly but consistently decreased pRb levels after oxaliplatin treatment in all cell types except oxaliplatin-resistant HCT116 cells.

### Effects of SLC1A1 knockdown and -overexpression on SN38- and oxaliplatin-induced cell death

**Fig. 6:**
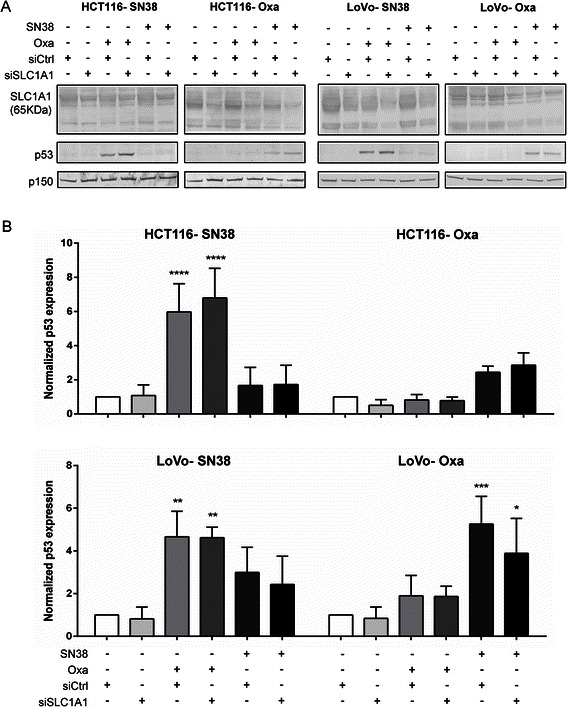
Effects of SLC1A1 siRNA on cell death and survival parameters after chemotherapy treatment. Drug-resistant HCT116 and LoVo cell lines were transfected with siRNA against SLC1A1 or corresponding mock siRNA (siCtrl.). 24 h later, cells were exposed to SN38 (0.8 μM), oxaliplatin (20 μM) as indicated, for 24 h. Equal amounts of protein per lane were separated by SDS-PAGE and the protein level of SLC1A1 and p53 was determined by Western blotting. **a** Representative Western blots, with p150 as loading control. **b** Densitometric quantifications of relative p53 protein level, based on 3 independent experiments per condition. Data are means with S.E.M. error bars of 3 independent experiments. *) *p* < 0.05, **) *p* < 0.01, ***) *p* < 0.001,****) *p* < 0.0001 compared to the control group without drug or TBOA treatment; Two-way ANOVA with Tukey post-test

DL-TBOA is a non-selective inhibitor of all SLC1A isoforms, thus the observed effects of DL-TBOA in the cells could potentially arise from its activity at SLC1A1, −A2, and/or -A6–7, whereas the lack of effect of UCPH-101 rules out the involvement of SLC1A3. We therefore asked whether p53 levels were similarly affected by siRNA-mediated SLC1A1 knockdown. About 50 % and 30 % SLC1A1 knockdown was obtained in resistant HCT116 and LoVo cell lines, respectively (Fig. 6). In LoVo, but not in HCT116 cells, SLC1A1 knockdown tended to reduce the oxaliplatin-induced increase in p53 protein level seen after DL-TBOA treatment, however, this effect was less marked than that seen after DL-TBOA treatment (compare with Fig. 5). Overexpression of SLC1A1 had no detectable effect on p53, p21, or PARP cleavage in any of the cell lines (Additional file 6: Figure S6).

### Effects of SN38-, oxaliplatin and DL-TBOA on subcellular localization of SLC1A1 in HCT116 cells

**Fig. 7:**
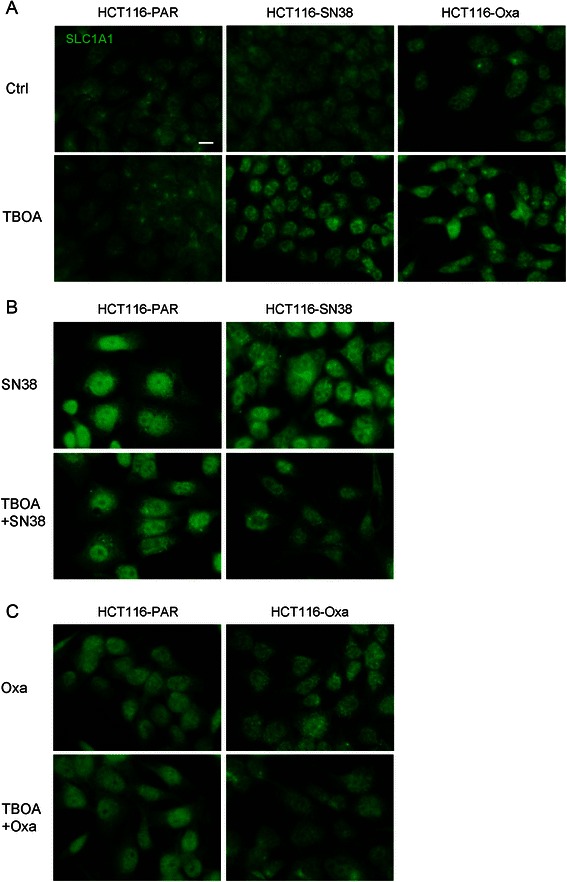
Subcellular localization of SLC1A1 in parental and resistant CRC cells – effects of chemotherapy and DL-TBOA. **a** Immunofluorescence images of parental (PAR), SN38 resistant and oxaliplatin resistant HCT116 cells treated or not for 48 h with 350 μM DL-TBOA, and stained with antibody against SLC1A1. **b** Parental and SN38-resistant HCT116 cells treated for 48 h with 0.8 μM SN38 in the absence or presence of 350 μM DL-TBOA, and stained as in A. **c** Parental and oxaliplatin-resistant HCT116 cells treated for 48 h with 20 μM oxaliplatin in the absence or presence of 350 μM DL-TBOA, and stained as in A. All conditions are representative of 2 or 3 independent biological replicates in duplicate. Scale bar: 10 μm. Additional file 7: Figure S7 shows the same images, merged with staining for DAPI and Rhodamine-conjugated phalloidin to visualize localization of nuclei and F-actin, respectively

To address the question of whether altered SLC1A1 localization was involved in the effects of SN38, oxaliplatin and DL-TBOA, we performed immunofluorescence analysis of the parental and resistant cell lines, in absence and presence of chemotherapeutics and DL-TBOA. SLC1A1 localization is shown in Fig. 7. In Additional file 7: Figure S7 the same images are shown merged with DAPI and F-actin staining. SLC1A1 is predominantly localized in intracellular vesicles, from where it undergoes regulated trafficking to the plasma membrane upon specific stimuli [34]. In agreement with this, SLC1A1 localized partially to the membrane and partially in a cytosolic compartment in both parental and SN38-resistant cells under control conditions (Fig. 7a). In contrast, in oxaliplatin-resistant cells, SLC1A1 staining was predominantly seen in the perinuclear/nuclear region under control conditions (Fig. 7a). In parental cells, treatment with SN38 or oxaliplatin induced a marked shift in SLC1A1 localization towards the perinuclear/nuclear region (Fig. 7b, c). In these cells, DL-TBOA had no detectable effect on SLC1A1 localization, either alone or in combination with the chemotherapeutic agents (Fig. 7a-c). In SN38-resistant cells, addition of DL-TBOA to the chemotherapeutic treatment increased the fraction of SLC1A1 fluorescence localized to the perinuclear/nuclear compartment, and a similar trend was seen with DL-TBOA alone (Fig. 7a, b). Notably, in oxaliplatin-resistant cells, a greater fraction of SLC1A1 was intracellular under control- and oxaliplatin-treated conditions, and this was partially reversed by DL-TBOA (Fig. 7a, c).

## Discussion

Resistance to irinotecan (of which SN38 is the active metabolite) and oxaliplatin is a major problem in CRC treatment, and the mechanisms of resistance remain incompletely understood. A major finding of this study is the striking difference between the effects of the glutamate transport inhibitor DL-TBOA on viability after SN38- and oxaliplatin treatment: DL-TBOA modestly exacerbated the loss of viability in untreated or SN38-treated cells, whereas it markedly counteracted oxaliplatin-induced cell loss. This suggests that glutamate transporter activity has a specific, negative impact on oxaliplatin-induced death, yet a modest positive effect on survival/growth in untreated and SN38-treated cells (which may also be present in oxaliplatin-treated cells but be masked by the strong, opposite effect).

### SLC1A1 expression and glutamate transporter activity are altered in SN38-resistant CRC cells

The SLC1A1 protein levels paralleled its mRNA levels, whereas SLC1A3 protein expression was not detectable. Basal glutamate transporter activity largely, but not completely, paralleled SLC1A1 expression and was inhibited by L-glutamate and by the broad glutamate transporter inhibitor DL-TBOA, but not by UCPH-101, a specific SLC1A3 inhibitor. Collectively, this suggests that SLC1A1 is at least partially responsible for the observed glutamate transporter activity. Although this is, to our knowledge, the first study to demonstrate SLC1A1 protein and activity changes in drug-resistant cancer cells, altered SLC1A1 mRNA expression was also reported in ovarian cancer cells and in the NCI-60 cancer cell line panel [14, 15], suggesting a more widespread relevance than CRC. In both HCT116 and LoVo cells, changes in [^3^H]-D-Asp uptake upon SN38 resistance development were quantitatively more pronounced than the changes in SLC1A1 protein level, and in oxaliplatin-resistant HCT116 cells, SLC1A1 expression was modestly increased, yet [^3^H]-D-Asp uptake modestly decreased. While not further addressed here, this suggests an additional role for posttranslational regulation of SLC1A1 activity, and/or, contributions from other SLC1A isoforms.

### Possible mechanisms involved in the effect of glutamate transporter inhibition on viability

We first hypothesized that the rescue of oxaliplatin-treated cells by DL-TBOA might reflect a dependence of oxaliplatin influx pathway(s) on glutamate transporter activity. The protein level of the major such pathway, CTR1, was modestly decreased by oxaliplatin treatment, yet was not regulated by DL-TBOA. Another possibility was that SLC1A1 might modulate cellular GSH levels, which also regulate oxaliplatin uptake via CTR1 [35]. SLC1A1 can directly transport l-cysteine and plays a major role in supporting GSH production [16, 19, 20], and also SLC1A1-mediated changes in extracellular glutamate signaling could contribute, under conditions where extracellular glutamate availability is a limiting factor. Multiple components of the glutamate signaling machinery are expressed in the colon epithelium, including, in addition to glutamate transporters [17], NMDA receptors (NMDARs) [36] and metabotropic glutamate receptors [37]. Indeed, NMDAR- and mGluR-antagonists inhibit the proliferation of HT29 cells [37]. On the other hand, excessive NMDAR activation induces increased [Ca^2+^]_i_ and consequent cell death in CRC cells [36]. Similarly, glutamate release autocrinely stimulates gliomas, yet is toxic to surrounding neurons [21–23]. Our microarray data [13] support the notion that glutamate- and glutathione homeostasis are broadly altered in the SN38- and oxaliplatin resistant CRC cells, in agreement with previous studies showing increased GSH levels and γ-GCS up-regulation in drug-resistant cancer cells [38]. Of note, the SN38-resistant LoVo cells, which exhibited a 4-fold increase in SLC1A1 expression, also show up-regulation of both mGluR and iGluR [13]. Also glutamate decarboxylase, which is rate-limiting for GABA production and was recently assigned important roles in small-cell lung cancer [39], shows increased expression in several of the resistant cell lines, as does the GSH-dependent ABCC2/MRP2 GS-X drug efflux pump [13]. Further supporting the notion that a change in basal GSH metabolism contributes to the resistant phenotype, cellular GSH was increased in SN38- and oxaliplatin-resistant HCT116, yet this did not correlate with the effects of DL-TBOA treatment on viability.

p53 induction by chemotherapeutic treatment was reduced by DL-TBOA in both HCT116 and LoVo cells. This differs from the opposite effects of DL-TBOA on viability in HCT116 and LoVo cells, thus, the specific mechanisms involved in the latter must be cell type-dependent and/or upstream of p53. Effects related to the cotransport of Na^+^ and H^+^ by the glutamate transporters may also be envisaged. Thus, in mouse astrocytes, glutamate uptake reduced cytosolic and mitochondrial pH and inhibited oxidative metabolism in a manner inhibited by DL-TBOA and only in cells expressing the glutamate transporters [40]. Other mechanisms previously implicated in oxaliplatin resistance include upregulation of Breast Cancer Resistance Protein (BCRP, ATPG2) and increased DNA-damage repair via up-regulation of Excision Repair Cross Complementing Protein 1 (ERCC1) [41], whereas SN38 resistance was proposed to involve down-regulation of topoisomerase-I [41]. Future studies should establish the possible link of these mechanisms to altered glutamate transporter activity.

Finally, the subcellular localization of SLC1A1 was altered in chemotherapy-resistant cells as well as by treatment with chemotherapy or DL-TBOA. Chemotherapy treatment (a reduction in viability), was associated with a shift of SLC1A1 towards a perinuclear/nuclear localization, except in HCT116-Oxa cells, in which SLC1A1 was perinuclear/nuclear already prior to treatment. Notably, the effects of DL-TBOA on SLC1A1 localization and cell viability correlated: In SN38-resistant cells, DL-TBOA augmented both SN38-induced loss of viability and chemotherapy-induced nuclear/perinuclear shift of SLC1A1, and in oxaliplatin-resistant cells, DL-TBOA counteracted both oxaliplatin-induced loss of viability and nuclear/perinuclear SLC1A1 localization. The mechanism involved cannot be deduced from the present studies, yet it is notable that regulated nuclear localization of SLC1A3 (GLAST-1) in cancer cells has been reported independently by two groups [18, 21]. Ye et al. [21] showed that SLC1A3 localized to the nucleus in glioma cells and glioblastoma patient brain tissue, but to the plasma membrane in normal astrocytes and normal brain tissue. Varini et al. [18] showed that nuclear localization of SLC1A3 was associated with reduced cell density/loss of cell-cell contacts. Neither study provided direct evidence to the mechanisms involved in this phenomenon, but if SLC1A1 localization is similarly regulated by cell-cell contacts, this might suggest that the translocation is downstream of reduced cell numbers in response to chemotherapy treatment, and also this interpretation is consistent with the precise correlation between the effect of DL-TBOA on viability and on SLC1A1 localization.

### Possible involvement of other excitatory amino acid transporters in the effects of DL-TBOA

The effect of DL-TBOA was concentration-dependent in the range congruent with known IC_50_-values for inhibition of SLC1A1, yet only a partially similar pattern was seen after siRNA knockdown of SLC1A1. It remains possible, therefore, that the effect of DL-TBOA involved other excitatory amino acid transporters than SLC1A1, and nonspecific effects can obviously not be excluded. However, the fact that DL-TBOA was always protective in oxaliplatin-treated, and always detrimental in SN38-treated, cells suggests that glutamate transporter inhibition impacts on a drug-specific, upstream mechanism, either at the level of drug influx/efflux, or, less likely, at the level of upstream interactions with the chromatin and associated DNA damage. Notably, public database information indicates that SLC1A1 is frequently down-regulated in CRC tumors (Additional file 1: Figure S1; www.oncomine.org; [42]). It is therefore tempting to speculate that in some CRC patients, this may confer a growth advantage similar to that exerted by DL-TBOA after oxaliplatin treatment. Future studies should assess excitatory amino acid transporter levels in CRC tumors from SN38- and oxaliplatin resistant and non-resistant patients.

## Conclusions

In conclusion, SLC1A1 expression and glutamate transporter activity are altered in SN38-resistant CRC cells, and the glutamate transporter inhibitor DL-TBOA reduces chemotherapy-induced p53 induction and augments CRC cell death induced by SN38, while strongly attenuating that induced by oxaliplatin. Our findings indicate that changes in glutamate transporter expression and activity may be relevant in CRC, diagnostically and in the context of choice of treatment regimen.

## Methods

### Reagents

SN38 was from Sigma-Aldrich, oxaliplatin from Sanofi-Aventis, and DL-Threo-β-Benzyloxyaspartic acid (DL-TBOA) from Tocris. UCPH101 was synthesized as described [29]. Primary antibodies were from Santa Cruz Biotechnology (SLC1A1, p21^WAF1/Cip1^ (p21) and CTR1), BD Transduction (p150), Cell Signaling Technology (poly-(ADP-ribose) polymerase-1 (PARP-1), phospho-Ser807/811-retinoblastoma protein, and p53). Rhodamine-phalloidin was from Invitrogen, and AlexaFluor488-conjugated secondary antibody from Life Technologies. 4',6-diamidino-2-phenylindole (DAPI) was from Invitrogen. Alkaline phosphatase-coupled secondary antibodies were from Sigma-Aldrich. [^3^H]-D-Aspartic acid ([^3^H]-D-Asp) was from PerkinElmer.

### Cell lines and treatments

HCT116 human CRC cells originate from a primary colon carcinoma [43], and LoVo cells from a metastatic nodule from a CRC patient [44]. Both cell lines are metastatic in xenograft models [45]. HCT116 cells were obtained from the NCI/Development Therapeutics Program, and LoVo cells from the American Tissue Culture Collection. The cell line identities of parental and resistant cell lines were confirmed using short tandem repeat DNA analysis (IdentiCell Cell Line Authentication Service, Aarhus University Hospital, Aarhus, Denmark). In addition, all cell lines were regularly assessed to be mycoplasma-free. HCT116 and LoVo sub-lines resistant to SN38 and oxaliplatin (hereafter denoted HCT116-SN38, HCT116-Oxa, LoVo-SN38 and LoVo-Oxa), respectively, were established by exposing parental HCT116 and LoVo cell lines to increasing doses of the respective chemotherapeutics for at least 45 passages [13]. Cells were grown in RPMI 1640 + Glutamax™, 10 % Foetal Bovine Serum (FBS), and 1 % Penicilin/Streptomyocin (Life Technologies) at 5 % CO_2_, 37 °C, and propagated by gentle trypsination every 3–4 days. Unless otherwise specified, experiments were carried out at 37 °C.

### Real-time quantitative PCR (qPCR) analysis

Total RNA was purified using the Machery-Nagel NucleoSpin® RNA II kit. cDNA was synthesized using SuperScript II RT (Invitrogen) and random primers. qPCR was carried out in triplicate in an ABI7900 Real Time PCR machine, using FastStart universal SYBR Green master mix (Roche Applied Bioscience), and 0.2 μM forward and reverse primers. Thermal profile was: 96 °C 10 min (96 °C 1 min, 60 °C 30 s, 72 °C 1 min) × 40. Normalization was done to GAPDH, PBGD and β-actin for LoVo, and PBGD and β-actin for HCT116. Primers were: SLC1A1:fw:5'-GGATGTCACCCTGATCATTGC-3', rv:5'-CCAAGGACGTTGACCATGGT-3; SLC1A3: fw:5'-CGAAGCCATCATGAGACTGGTA-3',rv:5'-TCCCAGCAATCAGGAAGAGAA-3'; β-actin: fw:5'-AGCGAGCATCCCCCAAAGT-3', rv:5'-GGGCACGAAGGCTCATCAT-3'; PBGD: fw:5'-TCCAAGCGGGAGCCATGTCTG-3', rv:5'-AGAATCTTGTCCCCTGTGGTGGA-3'; GAPDH: fw:5'-GAAGGTGAAGGTCGGAGTC-3, rv:5'-GAAGATGGTGATGGGATTTC-3'. Melting curves confirmed the presence of only one amplicon. Relative expression ratios were calculated as in [46].

### Immunoblotting

Cells were grown to a confluence of 60-80 % and treated with chemotherapy, siRNA- or plasmid transfection as indicated. Cells were washed once in PBS, lysed in lysis buffer (1 % SDS, 0.1 M Tris pH 7.5, and 1 mM Na_3_VO_4_), sonicated, and protein content determined (DC assay, BioRad). Lysates were mixed 2:1with NuPage LDS sample buffer (Invitrogen). SDS-PAGE was carried out in NOVEX chambers with NuPAGE 10 % Bis-Tris gels under reducing and denaturing conditions, using BenchMark protein ladder (Invitrogen), and 15 μg protein per lane. Proteins were transferred to PVDF membranes, which were Ponceau S stained, blocked for 1 h at 37 °C in 5 % nonfat dry milk in TBST (0.01 M Tris/HCl, 0.15 M NaCl, 0.1 % Tween 20, pH 7.4), incubated overnight at 4 °C with primary antibodies diluted in blocking buffer, washed in TBST, incubated with secondary antibodies for 1 h, washed in TBST, and developed using BCIP/NBT (KPL). Bands were scanned and quantified using UN-SCAN-IT (Silk Scientific).

### Cell viability assays

Cells were seeded at appropriate density (LoVo 10,000, and HCT116 5000 cells /100 μl) in growth medium in 96-well plates. Next day, cells were treated with inhibitors and/or chemotherapy in a total volume of 200 μl in growth medium and incubated for 48 h at 37 °C, 5 % CO_2_. The medium was replaced with 100 μl of 0.5 mg/ml 3-(4,5-dimethylthiazol-2-yl)-2,5-diphenyltetrazolium bromide (MTT) in growth medium. The reaction was stopped 1.5-2.5 h later by addition of 100 μl 20 % SDS in 0.02 M HCl, and incubation overnight to allow complete lysis of cells and formazan crystals. Absorbance was measured in an ELISA plate reader (Thermo Scientific MultiScan FC) at 550 nm. Data were background subtracted and % viability relative to controls calculated.

### Cell counting using the OPERA high throughput confocal

Cells were seeded in 96-well plates (Greiner Bio) one day prior to experiments. Cells were treated with chemotherapy and/or inhibitors as indicated and incubated for 48 h. Medium was aspirated, and cells were washed gently in ice cold PBS, fixed in 2 % paraformaldehyde, washed in PBS, and nuclei stained by 5 min incubation with DAPI. Plates were scanned using an OPERA high-throughput microscope (PerkinElmer). Nucleus counting was performed using proprietary OPERA software, based on 100 points per well, and cell counts from non-treated cells as controls.

### Constructs, siRNA and transfection

50 nM of non-specific siRNA (Eurofins MWG Operon, #10-5394-1/1) or SLC1A1 siRNA (GTGTTATATGCCACTAGGT; Mission siRNA, Sigma-Aldrich) was transfected using Lipofectamine 2000, into 40-50 % confluent LoVo and HCT116 cell lines. For overexpression, cells were transfected with full-length SLC1A1 in pcDNA3.1 [30] using 1 μg of DNA per well of a 6-well plate, and Lipofectamine 2000. 48 h after transfection, cells were treated with oxaliplatin or SN38 as indicated, and 24 h later, lysed for immunoblotting.

### [^3^H]-D-Asp uptake assay

The [^3^H]-D-Asp uptake assay was performed essentially as in [30]. Briefly, cells were split into poly-D-lysine-coated white 96-well plates (PerkinElmer). A similar number of cells were seeded for HCT116 parental, HCT116-SN38 and HCT116-Oxa cell lines (7 × 10^4^ cells/well) and LoVo parental, Lovo-SN38 and Lovo-Oxa cell lines (6 × 10^4^ cells/well). 16–24 h later, culture medium was aspirated, and cells were washed once with 100 μl assay buffer (Hank’s Buffered Saline Solution supplemented with 20 mM HEPES, 1 mM CaCl_2_ and 1 mM MgCl_2_, pH 7.4). 50 μl assay buffer supplemented with 100 nM [^3^H]-D-Asp and test compounds as indicated was added, and the plate was incubated at 37 °C for 6 min. Non-specific [^3^H]-D-Asp uptake was determined in wells with 3 mM L-glutamate. The assay mixture was quickly removed, and wells were washed with 2 × 100 μl ice-cold assay buffer, followed by 150 μl Microscint™20 scintillation fluid (PerkinElmer). The plate was shaken for 1 h and counted in a Wallac 1450 MicroBeta Trilux scintillation counter (GMI, Ramsey, MN).

### Measurement of cellular glutathione levels

Cells were seeded in 24-well plates (10^4^ cells per well), treated the next day with chemotherapy and/or DL-TBOA and incubated for 24 h. Medium was removed and cells were washed twice with ice-cold PBS, which was removed and 500 μl ice-cold 1 % Sulfosalicylic acid was added per well. Cells were incubated on ice for at least 10 min. After centrifugation (1 min, 15,000 g), 10 μl lysate was used to determine total GSx content. To measure GSSG content, 130 μl sample was mixed with 55 μl 0.2 M Tris (pH 9) and 5 μl 2-Vinylpyridine. Tubes were vortexed carefully and incubated for at least 1 h at room temperature. 10 μl of this mix was mixed first with 90 μl of water in a 96-well plate and then with a reaction mix (0.1 M sodium phosphate buffer pH 7.5 containing 1 mM EDTA, 10 mM NADPH, 10 mM DTNB and 0.05 μl Glutathione reductase, 2U/μl). Measurements were taken every 30 s for 10 min in an ELISA plate reader (Thermo Scientific MultiScan FC) at 405 nm absorbance. GSH values were obtained by subtraction of GSSG values from GSx values.

### Immunofluorescence analysis of SLC1A1

Immunofluorescence analysis was carried out essentially as in [47]. Cells grown on glass coverslips were fixed in 2 % paraformaldehyde, washed in TBST, permeabilized for 5 min (0.5 % Triton X-100 in TBST), blocked for 30 min in 5 % BSA in TBST, incubated with SLC1A1 primary antibody in TBST + 1 % BSA overnight at 4 °C, washed in TBST, and with Rhodamine-phalloidin and AlexaFluor488 conjugated secondary antibody (1:600 in TBS + 1 % BSA) for 1 h, followed by washing in TBST, and mounting in N-propyl-galleate mounting medium (2 % w/v in PBS/glycerol). DAPI was added for 3 min following incubation with the secondary antibody. Cells were visualized using the 60X/1.35 NA objective of an Olympus Bx63 epifluorescence microscope. No/negligible labeling was seen in the absence of primary antibody. Overlays were carried out using Adobe Photoshop software. No other image adjustment was performed.

### Statistical analysis

Statistical analysis was carried out in Graphpad Prism-6, using one-way ANOVA with Dunnett post-test, two-way ANOVA with Tukey post-test, or Students two-tailed *t-*test, as indicated.

### Ethics statement

No human material or human data except established cell lines and publically available information from the Oncomine database were used in the present study.

## Additional files

Below is the link to the electronic supplementary material.

Additional file 1: Figure S1. Microarray and Oncomine data showing the expression pattern of SLC1A1 and SLC1A3. (A) Microarray data showing the fold change in expression of SLC1A1 and SLC1A3 in SN38- and oxaliplatin (Oxa)-resistant HCT116 and LoVo cell lines compared to respective parental cell line. Data are from [13]. (B) The figure summarizes data from 15 different studies, showing the mRNA expression of SLC1A1 and SLC1A3 in CRC tissue relative to that in normal tissue. As seen, SLC1A1 was nearly ubiquitously downregulated, while the SLC1A3 level was generally unaltered. Data from Oncomine (www.oncomine.org; [26].

Additional file 2: Figure S2. Nucleus counting after treatment of parental and drug-resistant HCT116 and LoVo cells with DL-TBOA. Parental and drug-resistant HCT116 and LoVo cell lines seeded in 96-well dishes were exposed to SN38 (0.1 or 0.8 μM) or oxaliplatin (0.8 or 20 μM), alone or in combination with 70 or 350 μM DL-TBOA as indicated, for 48 h. Cells were washed in PBS, fixed in 2 % paraformaldehyde and nuclei were stained with DAPI. The number of adherent cells was determined by automated counting using an OPERA confocal microscope. (A-B) Parental HCT116 cells. (C) SN38 resistant HCT116 cells. (D) Oxaliplatin-resistant HCT116 cells. (E-F) Parental LoVo cells. (G) SN38 resistant LoVo cells. (H) Oxaliplatin-resistant LoVo cells. Data are means with S.E.M. error bars of 3 independent experiments. Values are normalized to those of untreated cells.

Additional file 3: Figure S3. Effects of DL-TBOA on cell death and survival parameters after chemotherapy treatment of HCT116 cells. Parental and drug-resistant HCT116 cell lines seeded in 6-well dishes were exposed to SN38 (0.8 μM) or oxaliplatin (20 μM), alone or in combination with 350 μM DL-TBOA as indicated, for 24 h. Equal amounts of protein per lane were separated by SDS-PAGE and the protein levels of p21, and PARP-1 (full-length and cleaved, the latter indicated by arrowheads) were determined by Western blotting. Top: Representative Western blots, with p150 as loading control. Bottom: Densitometric quantifications based on 3 independent experiments per condition. Data are means with S.E.M. error bars of 3 independent experiments. *) *p* < 0.05, **) *p* < 0.01, ***) *p* < 0.001,****) *p* < 0.0001 compared to the control group without drug or TBOA treatment; #) *p* < 0.05 compared to controls without TBOA treatment. Two-way ANOVA with Tukey post-test.

Additional file 4: Figure S4. Effects of DL-TBOA on cell death and survival parameters after chemotherapy treatment of LoVo cells. Parental and drug-resistant LoVo cell lines seeded in 6-well dishes were exposed to SN38 (0.8 μM) or oxaliplatin (20 μM), alone or in combination with 350 μM DL-TBOA as indicated, for 24 h. Equal amounts of protein per lane were separated by SDS-PAGE and the protein levels of p21, and PARP-1 (full-length and cleaved, the latter indicated by arrowheads) were determined by Western blotting. Top: Representative Western blots, with p150 as loading control. Bottom: Densitometric quantifications based on 3 independent experiments per condition. Data are means with S.E.M. error bars of 3 independent experiments. *) *p* < 0.05, **) *p* < 0.01, ***) *p* < 0.001,****) *p* < 0.0001 compared to the control group without drug or TBOA treatment; Two-way ANOVA with Tukey post-test.

Additional file 5: Figure S5. Effects of DL-TBOA on proliferation after chemotherapy treatment. Parental and drug-resistant HCT116 (A) and LoVo (B) cell lines seeded in 6-well dishes were exposed to SN38 (0.8 μM) or oxaliplatin (20 μM) alone or in combination with 350 μM DL-TBOA as indicated, for 24 h. Equal amounts of protein per lane were separated by SDS-PAGE and the protein levels of phosphorylation of retinoblastoma protein on Ser 807/811 (pRb) were determined by Western blotting. Top: Representative Western blots, with β-actin as loading control. Bottom: Densitometric quantifications based on 3 independent experiments per condition. Data are means with S.E.M. error bars of 3 independent experiments. Two-way ANOVA with Tukey post- test.

Additional file 6: Figure S6. SLC1A1 overexpression has no detectable effect on cell death- and survival parameters tested. Representative blot data of 3 independent experiments of HCT116 (A) or LoVo (B) cell lines, in absence or presence of transient overexpression of wild type SLC1A1 (pSLC1A1) or corresponding empty vector (pcDNA3.1), followed by 24 h of chemotherapeutic treatment (0.8 μM SN38 or 20 μM Oxa). The protein levels of SLC1A1, p53, p21, and PARP-1 (full-length and cleaved, the latter indicated by arrowheads) were determined by Western blotting. p150 is shown as a loading control.

Additional file 7: Figure S7. Subcellular localization of SLC1A1, nuclei, and F-actin in parental and resistant CRC cells-effects of chemotherapy and DL-TBOA. (A) Immunofluorescence images of parental (PAR), SN38 resistant and oxaliplatin resistant HCT116 cells treated or not for 48 h with 350 μM DL-TBOA, and stained with antibody against SLC1A1 (green) and with DAPI (blue) and Rhodamine-conjugated phalloidin (red) to visualize localization of nuclei and F-actin, respectively. (B) Parental and SN38-resistant HCT116 cells treated for 48 h with 0.8 μM SN38 in the absence or presence of 350 μM DL-TBOA, and stained as in A. (C) Parental and oxaliplatin-resistant HCT116 cells treated for 48 h with 20 μM oxaliplatin in the absence or presence of 350 μM DL-TBOA, and stained as in A. All conditions are representative of 2 or 3 independent biological replicates in duplicate. Scale bar: 10 μm.

## Abbreviations

CRC: Colorectal cancer
CTR1: Cu^2+^-transporter-1
EAAT: Excitatory amino acid transporter
GSH: Reduced glutathione, PARP, poly-ADP ribose
pRb: Phospho-Retinoblastomaprotein
SLC: Solute carrier
DL-TBOA: DL-Threo-β-Benzyloxyaspartic acid
