# Supplementary figures and images for: The glutamate transport inhibitor DL-Threo-β-Benzyloxyaspartic acid (DL-TBOA) differentially affects SN38- and oxaliplatin-induced death of drug-resistant colorectal cancer cells
Source: BMC Cancer. 2015 May 16;15:411. doi: 10.1186/s12885-015-1405-8 (PMC4445981; doi:10.1186/s12885-015-1405-8)

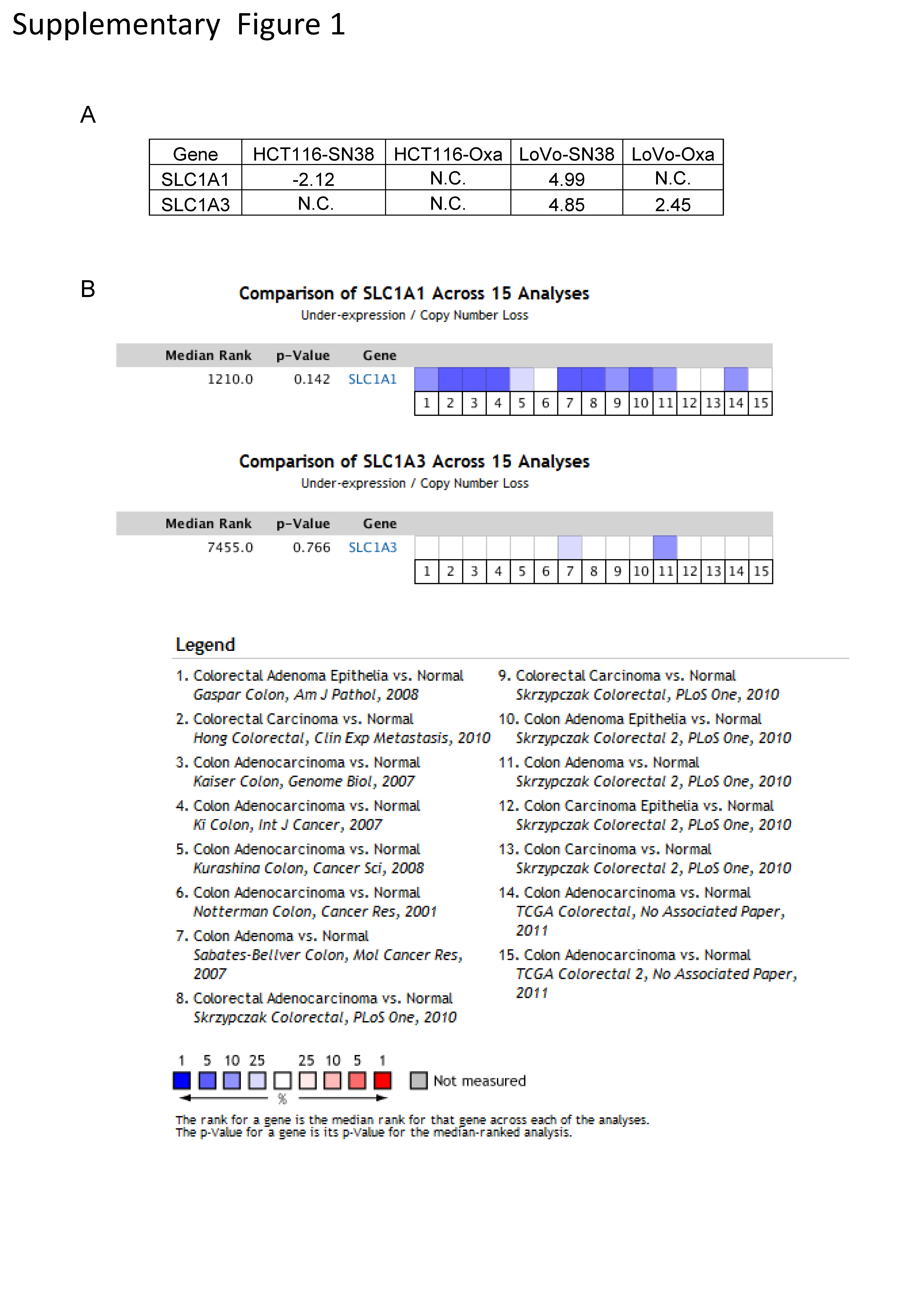

Supplement: Supplementary file 1 — Microarray and Oncomine data showing the expression pattern of SLC1A1 and SLC1A3. (A) Microarray data showing the fold change in expression of SLC1A1 and SLC1A3 in SN38- and oxaliplatin (Oxa)-resistant HCT116 and LoVo cell lines compared to respective parental cell line. Data are from [13]. (B) The figure summarizes data from 15 different studies, showing the mRNA expression of SLC1A1 and SLC1A3 in CRC tissue relative to that in normal tissue. As seen, SLC1A1 was nearly ubiquitously downregulated, while the SLC1A3 level was generally unaltered. Data from Oncomine (www.oncomine.org; [26]. [file 12885_2015_1405_MOESM1_ESM.tiff]

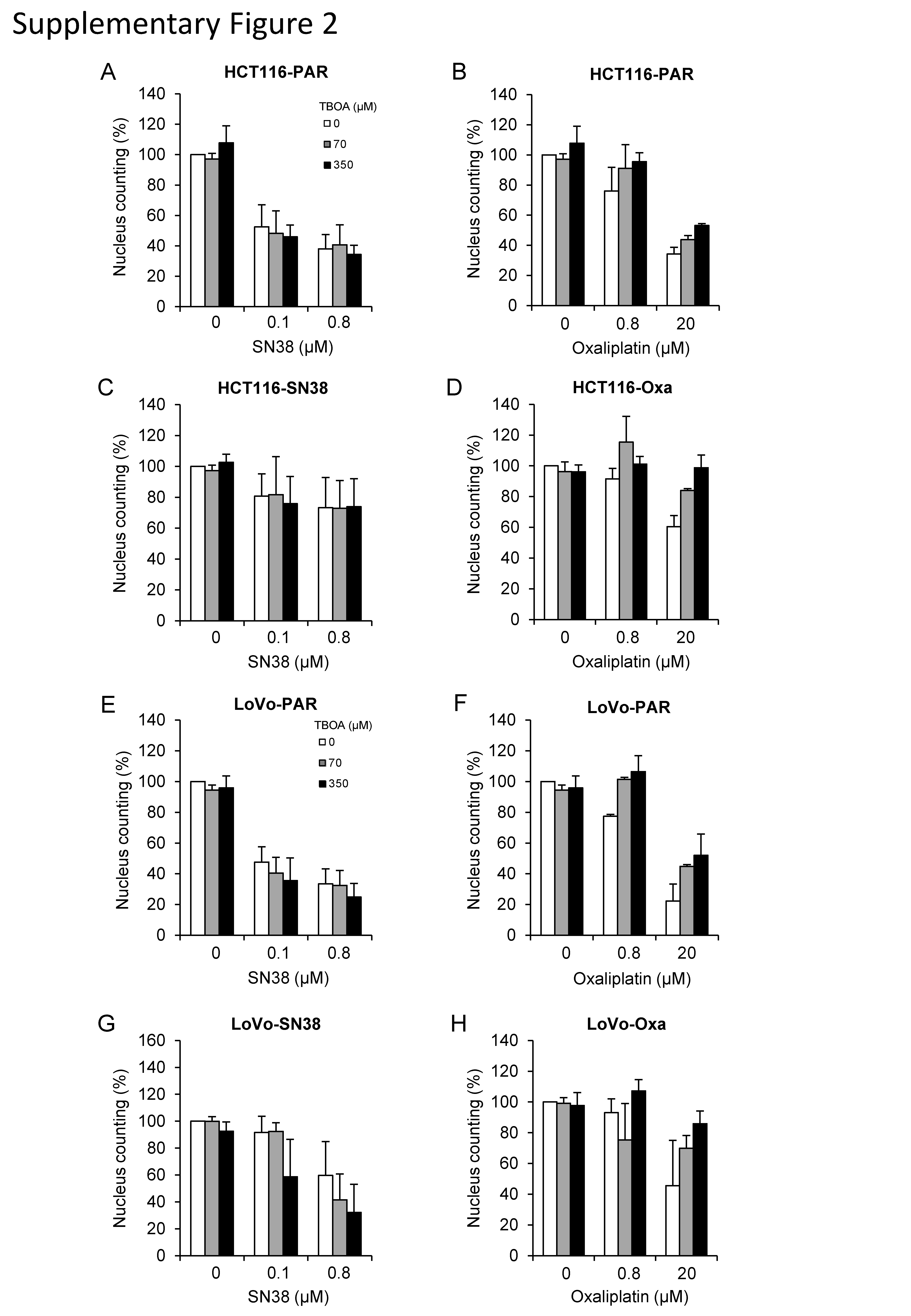

Supplement: Supplementary file 2 — Nucleus counting after treatment of parental and drug-resistant HCT116 and LoVo cells with DL-TBOA. Parental and drug-resistant HCT116 and LoVo cell lines seeded in 96-well dishes were exposed to SN38 (0.1 or 0.8 μM) or oxaliplatin (0.8 or 20 μM), alone or in combination with 70 or 350 μM DL-TBOA as indicated, for 48 h. Cells were washed in PBS, fixed in 2 % paraformaldehyde and nuclei were stained with DAPI. The number of adherent cells was determined by automated counting using an OPERA confocal microscope. (A-B) Parental HCT116 cells. (C) SN38 resistant HCT116 cells. (D) Oxaliplatin-resistant HCT116 cells. (E-F) Parental LoVo cells. (G) SN38 resistant LoVo cells. (H) Oxaliplatin-resistant LoVo cells. Data are means with S.E.M. error bars of 3 independent experiments. Values are normalized to those of untreated cells. [file 12885_2015_1405_MOESM2_ESM.tiff]

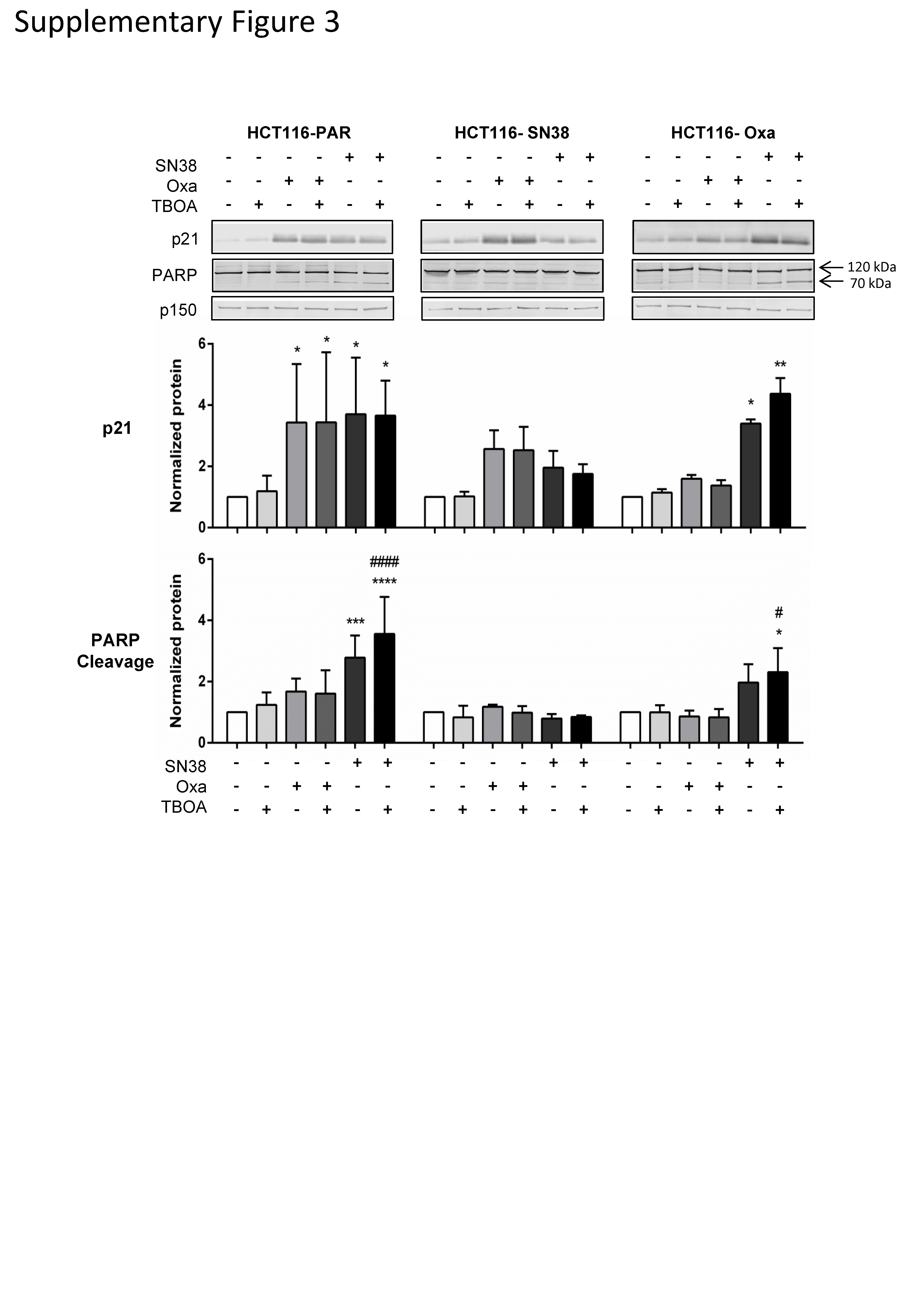

Supplement: Supplementary file 3 — Effects of DL-TBOA on cell death and survival parameters after chemotherapy treatment of HCT116 cells. Parental and drug-resistant HCT116 cell lines seeded in 6-well dishes were exposed to SN38 (0.8 μM) or oxaliplatin (20 μM), alone or in combination with 350 μM DL-TBOA as indicated, for 24 h. Equal amounts of protein per lane were separated by SDS-PAGE and the protein levels of p21, and PARP-1 (full-length and cleaved, the latter indicated by arrowheads) were determined by Western blotting. Top: Representative Western blots, with p150 as loading control. Bottom: Densitometric quantifications based on 3 independent experiments per condition. Data are means with S.E.M. error bars of 3 independent experiments. *) p < 0.05, **) p < 0.01, ***) p < 0.001,****) p < 0.0001 compared to the control group without drug or TBOA treatment; #) p < 0.05 compared to controls without TBOA treatment. Two-way ANOVA with Tukey post-test. [file 12885_2015_1405_MOESM3_ESM.tiff]

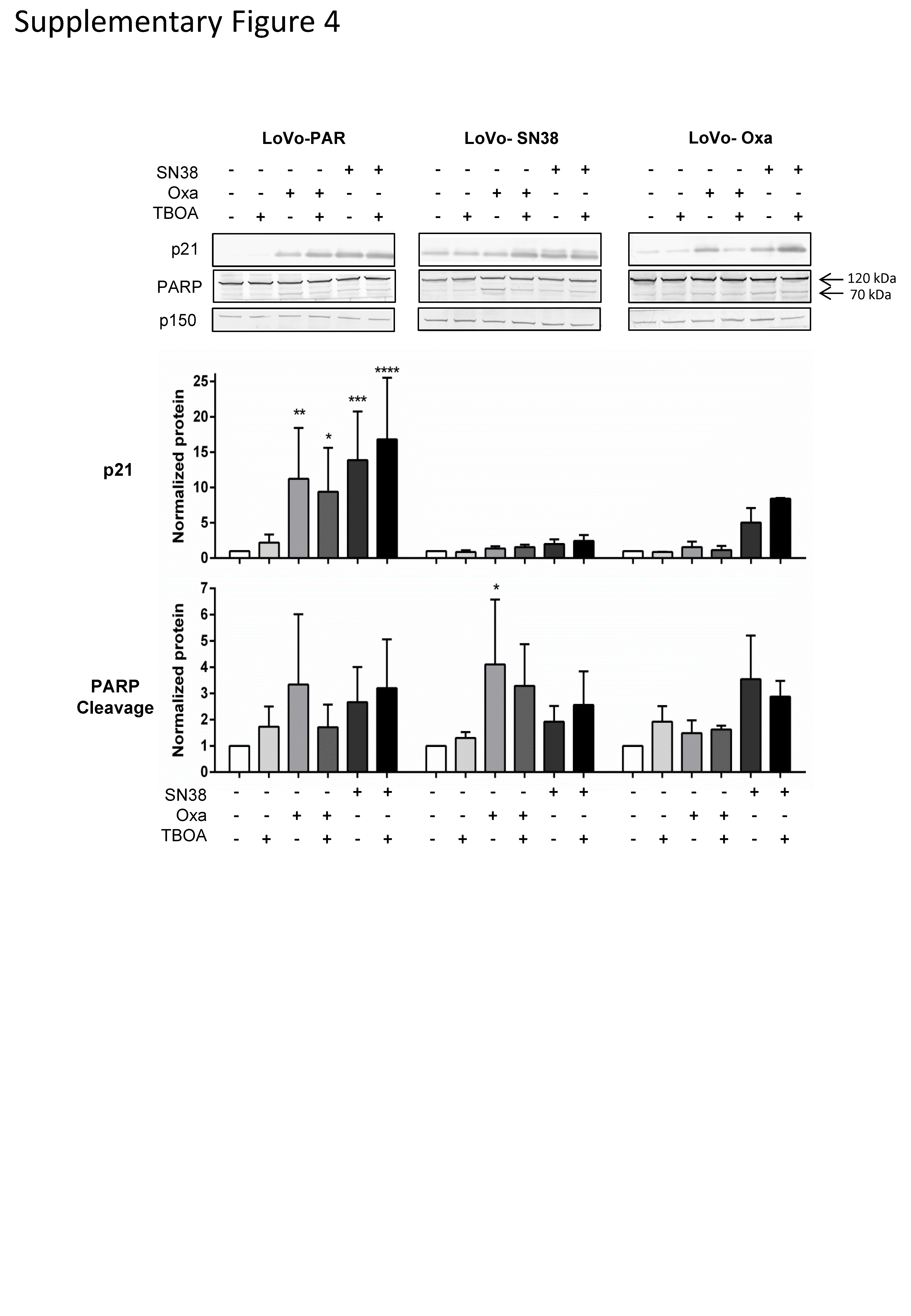

Supplement: Supplementary file 4 — Effects of DL-TBOA on cell death and survival parameters after chemotherapy treatment of LoVo cells. Parental and drug-resistant LoVo cell lines seeded in 6-well dishes were exposed to SN38 (0.8 μM) or oxaliplatin (20 μM), alone or in combination with 350 μM DL-TBOA as indicated, for 24 h. Equal amounts of protein per lane were separated by SDS-PAGE and the protein levels of p21, and PARP-1 (full-length and cleaved, the latter indicated by arrowheads) were determined by Western blotting. Top: Representative Western blots, with p150 as loading control. Bottom: Densitometric quantifications based on 3 independent experiments per condition. Data are means with S.E.M. error bars of 3 independent experiments. *) p < 0.05, **) p < 0.01, ***) p < 0.001,****) p < 0.0001 compared to the control group without drug or TBOA treatment; Two-way ANOVA with Tukey post-test. [file 12885_2015_1405_MOESM4_ESM.tiff]

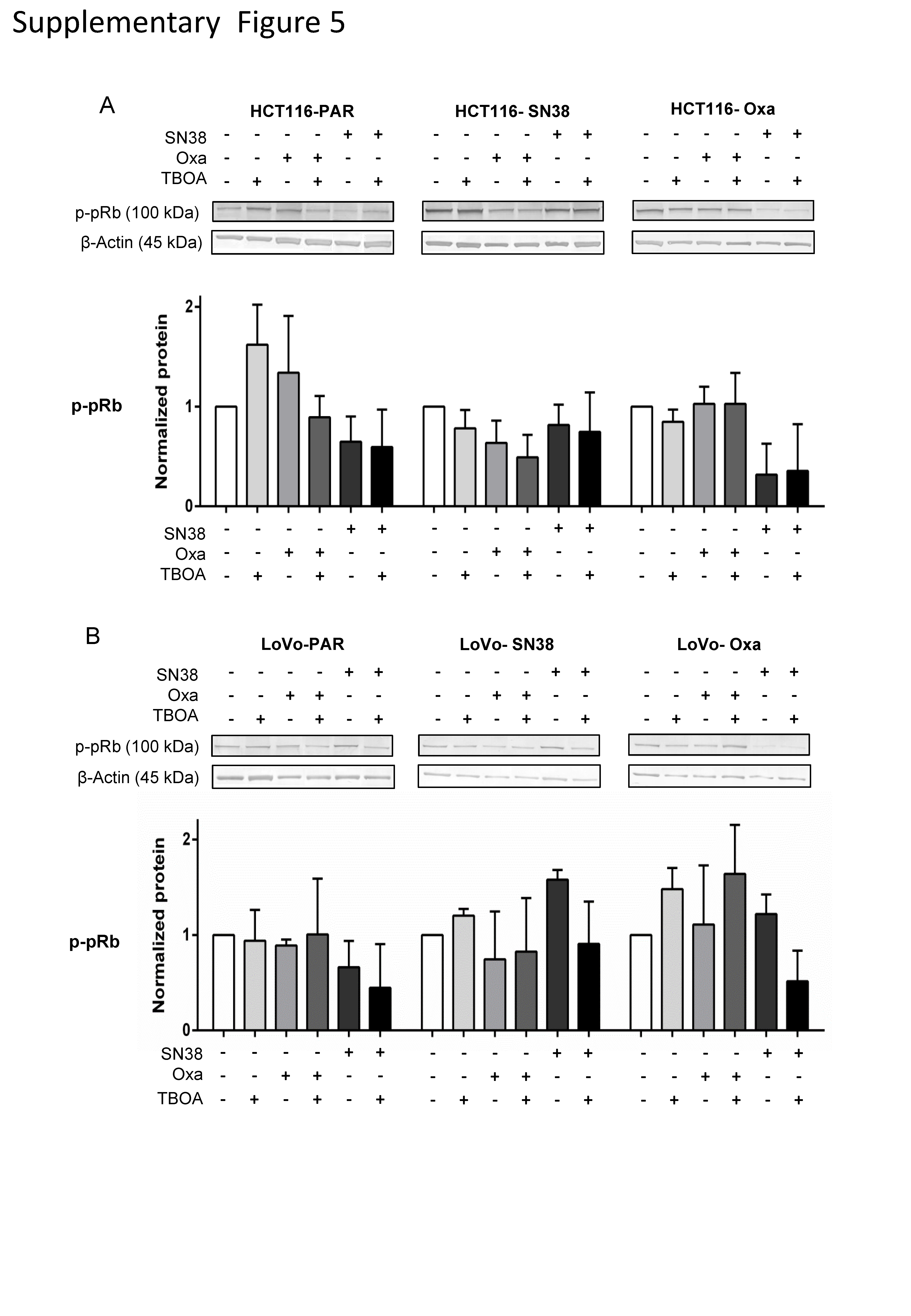

Supplement: Supplementary file 5 — Effects of DL-TBOA on proliferation after chemotherapy treatment. Parental and drug-resistant HCT116 (A) and LoVo (B) cell lines seeded in 6-well dishes were exposed to SN38 (0.8 μM) or oxaliplatin (20 μM) alone or in combination with 350 μM DL-TBOA as indicated, for 24 h. Equal amounts of protein per lane were separated by SDS-PAGE and the protein levels of phosphorylation of retinoblastoma protein on Ser 807/811 (pRb) were determined by Western blotting. Top: Representative Western blots, with β-actin as loading control. Bottom: Densitometric quantifications based on 3 independent experiments per condition. Data are means with S.E.M. error bars of 3 independent experiments. Two-way ANOVA with Tukey post- test. [file 12885_2015_1405_MOESM5_ESM.tiff]

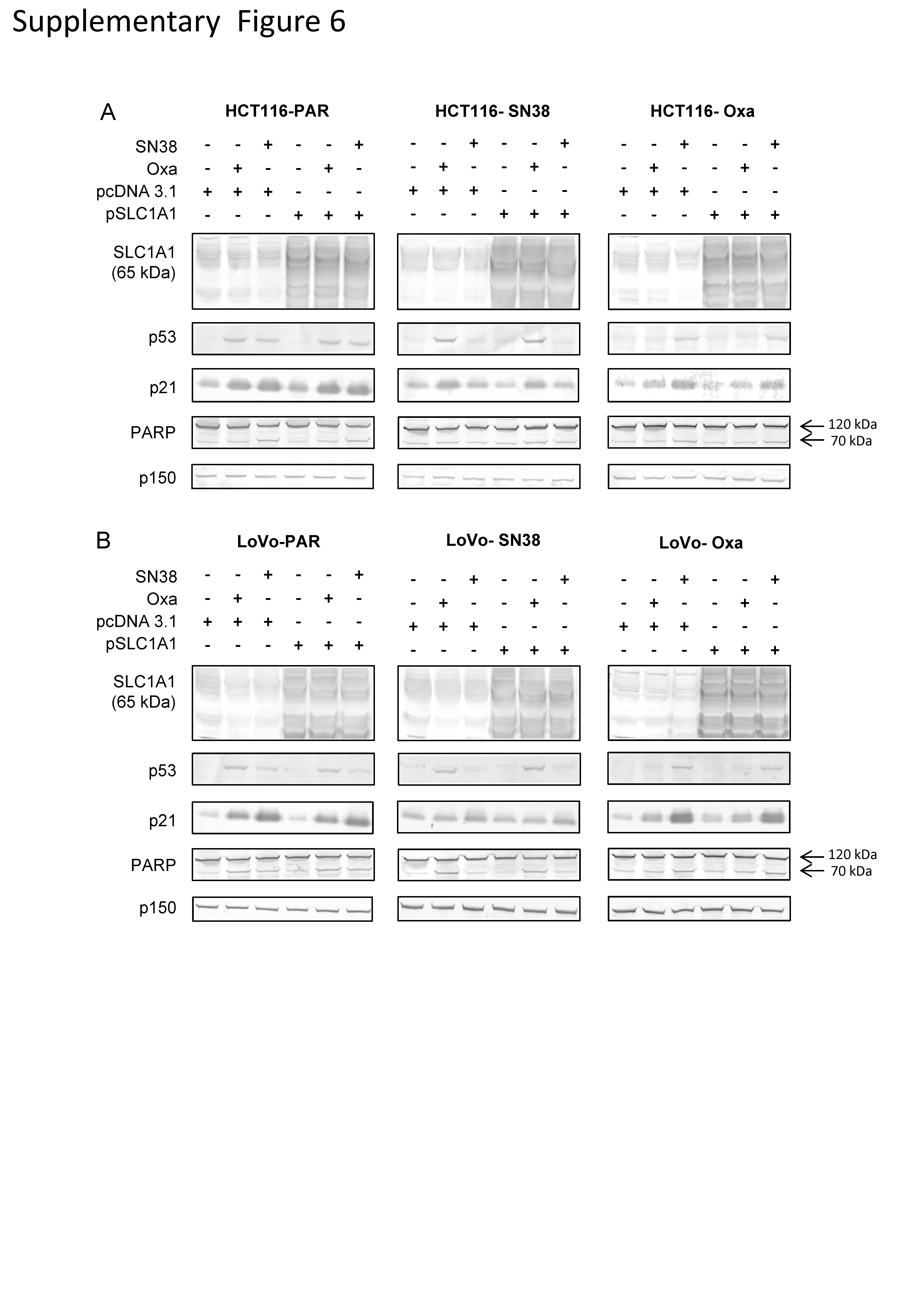

Supplement: Supplementary file 6 — SLC1A1 overexpression has no detectable effect on cell death- and survival parameters tested. Representative blot data of 3 independent experiments of HCT116 (A) or LoVo (B) cell lines, in absence or presence of transient overexpression of wild type SLC1A1 (pSLC1A1) or corresponding empty vector (pcDNA3.1), followed by 24 h of chemotherapeutic treatment (0.8 μM SN38 or 20 μM Oxa). The protein levels of SLC1A1, p53, p21, and PARP-1 (full-length and cleaved, the latter indicated by arrowheads) were determined by Western blotting. p150 is shown as a loading control. [file 12885_2015_1405_MOESM6_ESM.tiff]

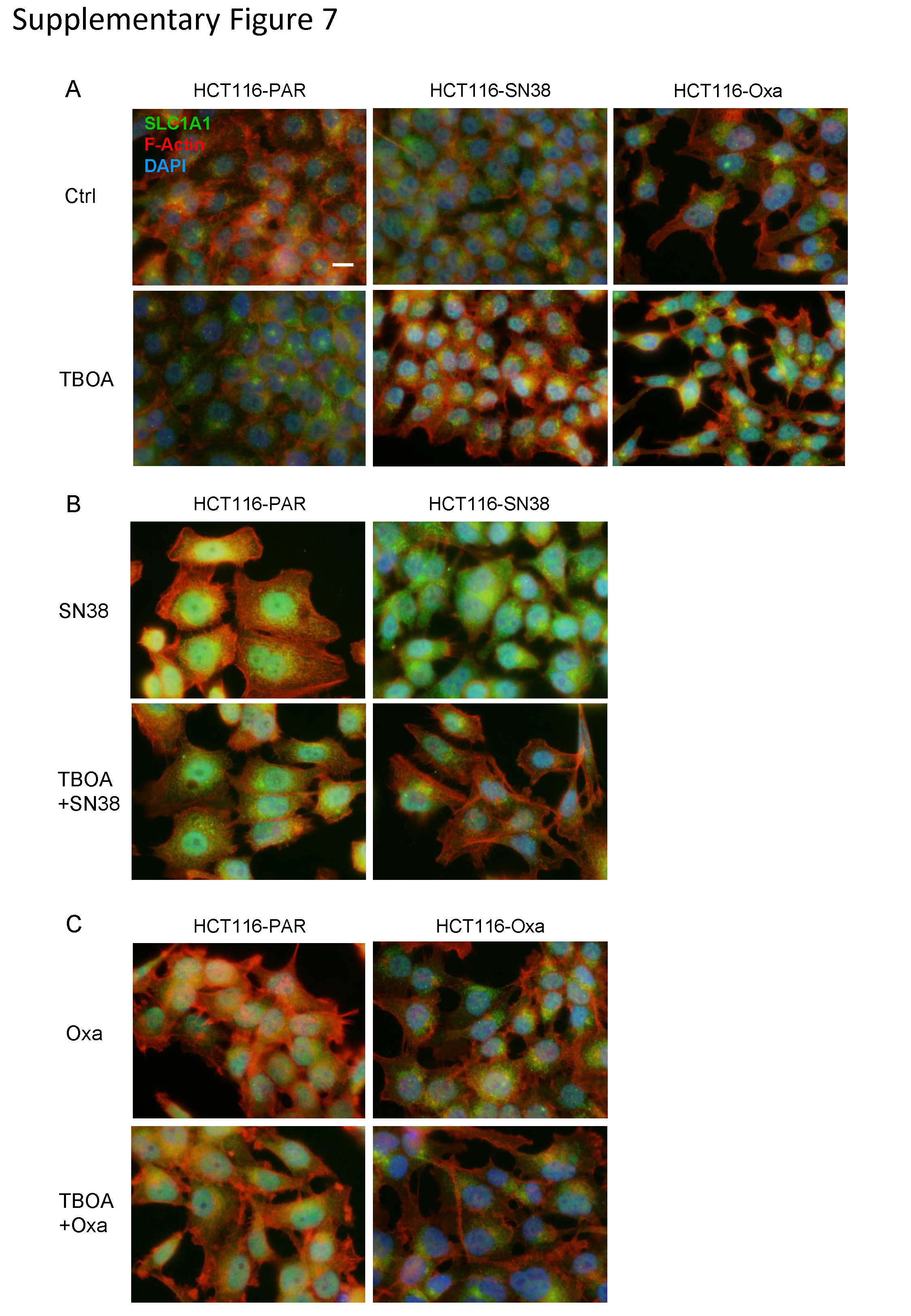

Supplement: Supplementary file 7 — Subcellular localization of SLC1A1, nuclei, and F-actin in parental and resistant CRC cells-effects of chemotherapy and DL-TBOA. (A) Immunofluorescence images of parental (PAR), SN38 resistant and oxaliplatin resistant HCT116 cells treated or not for 48 h with 350 μM DL-TBOA, and stained with antibody against SLC1A1 (green) and with DAPI (blue) and Rhodamine-conjugated phalloidin (red) to visualize localization of nuclei and F-actin, respectively. (B) Parental and SN38-resistant HCT116 cells treated for 48 h with 0.8 μM SN38 in the absence or presence of 350 μM DL-TBOA, and stained as in A. (C) Parental and oxaliplatin-resistant HCT116 cells treated for 48 h with 20 μM oxaliplatin in the absence or presence of 350 μM DL-TBOA, and stained as in A. All conditions are representative of 2 or 3 independent biological replicates in duplicate. Scale bar: 10 μm. [file 12885_2015_1405_MOESM7_ESM.tiff]
